# Enhancing the potency of CAR-T cells against solid tumors through transcription factor engineering

**DOI:** 10.1172/jci.insight.193048

**Published:** 2025-07-22

**Authors:** Ruoqi Chen, Lianqing Chen, Yu Tang, Xiaolin Shen, Yajie Wang, Peng Tang, Xingchao Shentu, Jie Sun

**Affiliations:** 1 Eye Center of Second Affiliated Hospital, School of Medicine, Zhejiang University, Zhejiang Provincial Key Laboratory of Ophthalmology, Zhejiang Provincial Clinical Research Center for Eye Diseases. Zhejiang Provincial Engineering Institute on Eye Diseases, Hangzhou, China.; 2 Liangzhu Laboratory, Zhejiang University, Hangzhou, China.; 3 Bone Marrow Transplantation Center of the First Affiliated Hospital and Department of Cell Biology, Zhejiang University School of Medicine, Hangzhou, China.; 4 Institute of Hematology, Zhejiang University & Zhejiang Engineering Laboratory for Stem Cell and Immunotherapy, Hangzhou, China.; 5 Department of Orthopedic Surgery of Second Affiliated Hospital, Zhejiang University School of Medicine, Hangzhou, China.

## Abstract

Transcription factors (TFs) play a pivotal role in the development and differentiation of T cells. Recent studies have highlighted unique transcriptional profiles in chimeric antigen receptor T (CAR-T) cells derived from patients with favorable clinical outcomes, suggesting a potential link between TF modulation and improved therapeutic efficacy. Although CAR-T cell therapies have shown some success in treating hematological malignancies, they are limited by challenges such as poor persistence, functional exhaustion, and tumor resistance. To overcome these limitations, researchers have attempted to enhance the efficacy of CAR-T cells through manipulation of TF expression. This Review provides a comprehensive overview of TF engineering in CAR-T cells and elucidates the complex regulatory network between TFs. Notably, modification of basic leucine zipper ATF-like transcription factor in CAR-T cells results in contradictory functional outcomes in different studies. We summarize the potential factors leading to such results and elucidate the importance of setting up a relevant in vitro model to evaluate the effect of TFs on CAR-T cells. In conclusion, this Review highlights the latest advances in TF modifications and proposes strategies for harnessing these insights to empower CAR-T cells with superior antitumor efficacy.

## Introduction

Chimeric antigen receptor T (CAR-T) cell therapy is a promising immunotherapy that has achieved great success in treating hematological malignancies (1). To date, seven CAR-T cell products have been approved by the US Food and Drug Administration (FDA), including 5 CD19-specific CAR-T cell products and 2 products that target B cell maturation antigen (2, 3). However, the efficacy of CAR-T cell therapy in solid tumors remains limited, primarily attributed to CAR-T cell dysfunction characterized by loss of effector function, poor persistence, and exhaustion during chronic antigen exposure (4–7). To overcome these limitations, researchers have explored various strategies, and manipulation of transcription factors (TFs) has shown great potential.

TFs are DNA-binding proteins that regulate gene transcription (4). Recent advances in sequencing technologies have delineated the unique transcriptional features of CAR-T cells both in vitro and in vivo (8–10). These studies have identified several critical TFs that participate in T cell development and differentiation, and modification of these TFs through KO or overexpression strategies has demonstrated exciting outcomes (8, 11–16). CRISPR/Cas9-mediated KO of exhaustion-related TFs alleviates CAR-T cell dysfunction (9, 11, 17), while overexpression of memory-related TFs promotes memory-like features (12, 13, 18). This Review aims to provide a comprehensive overview of TF manipulation of CAR-T cells, focusing on the regulatory network that governs their functions.

## Different subsets of CAR-T cells in cancer immunotherapy

CAR-T cells are genetically engineered T cells expressing CARs for tumor targeting (19). Based on the characteristics of T cells, they can be divided into several subsets.

Naive T cells are defined as immature T cells that have not been antigen-triggered. Interaction with antigens via the TCR or CAR structure activates naive T cells, resulting in rapid expansion and differentiation into effector or memory T cells (20–22). Effector T cells display superior tumor-killing capacity compared with other T cell subsets. During acute infections, most effector T cells die after the elimination of antigens, while a small subset transforms into memory T cells. However, upon continuous antigen exposure in chronic infections and cancer, effector T cells gradually become functionally exhausted, characterized by reduced cytokine production (IL-2, IFN-γ, and TNF-α), upregulated inhibitory receptors (programmed cell death 1 [PD-1], T cell immunoglobulin mucin receptor 3 [TIM-3], and lymphocyte activation gene 3 [LAG-3]), and alterations in their transcriptional and epigenetic profiles (20, 21, 23). Analysis of patients receiving CAR-T cell therapy revealed that patients with limited efficacy exhibited a larger proportion of exhausted T cells rather than effector T cells (24). Therefore, approaches to reverse T cell exhaustion in CAR-T therapy are of critical importance.

Memory T cells, with a long lifespan and capacity for self-renewal, can be further divided into several subsets, including central memory (Tcm), effector memory T (Tem), and circulating stem cell memory T (Tscm) cells (23, 25). CAR-T cells exhibiting memory phenotypes are more likely to yield long-term treatment outcomes (26–30). Several studies have revealed the transcriptional and epigenetic regulation mechanisms driving T cell memory differentiation, paving the way for TF engineering to achieve long-term persistence of CAR-T cells with less differentiated phenotypes (12, 31).

## TF manipulation to overcome CAR-T cell exhaustion

**Figure 1:**
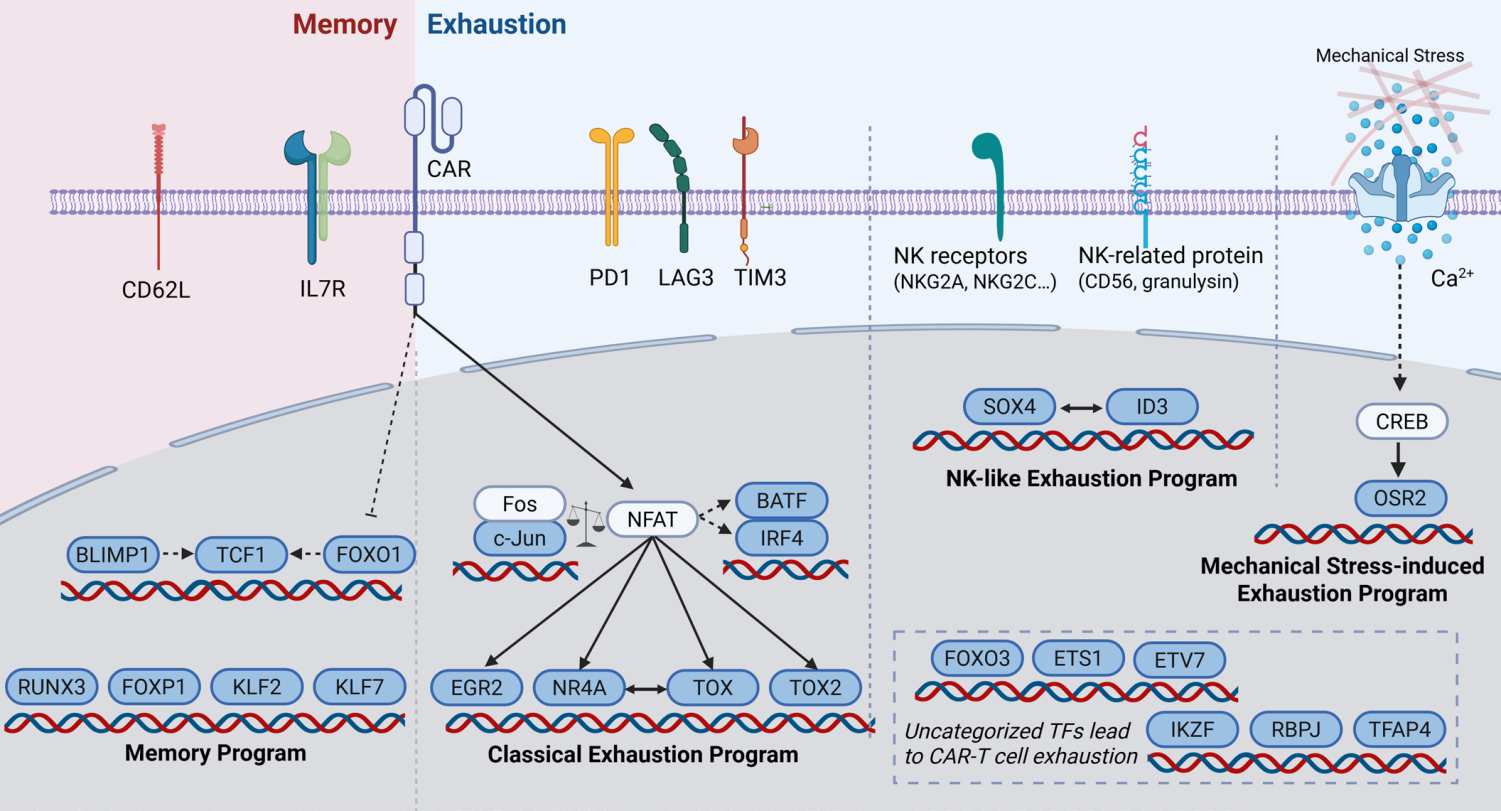
Overview of the transcriptional regulatory networks that drive CAR-T cell differentiation into exhaustion and memory states. A complex transcriptional program regulates CAR-T cell plasticity. Memory differentiation is driven by the regulation of FOXO1 and BLIMP and the downstream TCF1. RUNX3, FOXP1, KLF2, and KLF7 also promote memory T cell differentiation by activating the memory program. The canonical exhaustion pathway involves the NFAT-TOX/NR4A axis. NFAT can also cooperate with BATF, IRF4, and EGR2. CAR-T cells with a 4-1BB costimulatory domain exhibit a NK-like exhausted phenotype via the regulation of ID3 and SOX4. In addition, mechanical stress induces T cell exhaustion through the CREB/OSR2 pathway. IL7R, interleukin-7 receptor; LAG3, lymphocyte activation gene 3.

Chronic tumor antigen stimulation drives T cell exhaustion, which is characterized by a distinct transcriptomic profile (20, 21, 23). Researchers have identified specific TFs that regulate exhaustion via different mechanisms. Modification of these TFs in TCR-T or CAR-T cells can successfully alleviate T cell exhaustion (32, 33), demonstrating promising potential for clinical translation (Figure 1 and Tables 1 and 2) (8, 9, 11, 15, 16, 31, 34–36).

## TFs within the classical NFAT-TOX/NR4A axis

The process of T cell activation and differentiation is initiated by the interaction of nuclear factor of activated T cells (NFAT) with activator protein 1 (AP-1) (37). AP-1 is a family of heterodimeric TFs containing proteins typically formed by Jun proto-oncogene (c-Jun) and c-Fos. As the binding partner of AP-1, NFAT also has the capacity to bind to other TFs, including thymocyte selection associated high mobility group box (TOX) and nuclear receptor subfamily 4 group A (NR4A) (33, 38). Numerous studies have validated that the equilibrium between NFAT and AP-1 is pivotal for T cell states. The relative deficiency of AP-1 enables unbound NFAT to interact with TOX and NR4A, consequently inducing T cell exhaustion rather than the effector program, commonly referred to as the classical TCR-NFAT-TOX/NR4A pathway (39). Basic leucine zipper ATF-like (BATF) can also influence this axis by binding to the AP-1–interferon regulatory factor 4 (IRF4) composite elements (AICEs) (11). The manipulation of TFs within this pathway has been demonstrated to effectively reduce T cell exhaustion, as detailed below (Table 1).

### c-Jun.

c-Jun is an AP-1 family TF that forms a heterodimer with Fos. During chronic antigen stimulation, decreased c-Jun-Fos heterodimer persistence leads to excessive NFAT and in turn triggers the downstream exhaustion program (40). A study generated anti-GD2 CAR-T cells with an E101K mutation in the scFv region (HA-28z), which displayed a more severe exhaustion phenotype (8). Analyzing the exhaustion transcriptional program of HA-28z CAR-T cells, researchers recognized an overabundance of AP-1-IRF complexes, leading to a functional deficiency in activating AP-1 Fos-Jun heterodimers (8). Overexpression of c-Jun can reverse the dysfunction in exhausted HA-28z CAR-T cells, resulting in enhanced antitumor efficacy through elevated IL-2 and IFN-γ production, sustained proliferation, and increased fraction of Tscm and Tcm subsets (8). Another study on glypican-2 antigen-targeting CAR-T cells also demonstrated that c-Jun overexpression can improve neuroblastoma control, with robust expansion and prolonged persistence (41). Interestingly, c-Jun overexpression can also lower the antigen threshold for CAR-T cells (41). Given that some CAR-T therapy failures are due to low antigen expression on target cells, c-Jun overexpression to increase antigen sensitivity could be clinically significant. In a phase I trial (NCT05274451), receptor tyrosine kinase-like orphan receptor 1–targeted CAR-T cells with c-Jun overexpression demonstrated antiexhaustion properties and enhanced stemness for solid tumor treatment, although 1 fatal respiratory failure event warrants further on-target/off-tumor toxicity evaluation (42).

Concurrently, another phase I trial (NCT04835519) achieved clinical efficacy in patients with relapsed/refractory acute myeloid leukemia, yet safety profile remains a critical concern (43). Three patients developed grade 1–2 cytokine release syndrome (CRS), and one experienced grade 4 CRS (43). Additionally, neurotoxicity and graft-versus-host disease (GVHD) were observed (43). These preliminary findings underscore the need for further investigation of the therapeutic potential and toxicities of c-Jun–overexpressing CAR-T cells.

### TOX and TOX2.

TFs TOX and TOX2 have been identified as downstream targets of NFAT (33). Unbound NFAT directly upregulates TOX, sustained expression of which can lead to T cell exhaustion (33, 40). Seo et al. identified TOX and TOX2 upregulation in PD-1^+^TlM-3^hi^ exhausted CAR-T cells in a B16–human CD19 (hCD19) melanoma model, whereas double KO of TOX and TOX2 revitalized the exhaustion process and restored effector functions, including elevated IFN-γ and TNF-α production (33). Intriguingly, compared with single KOs, the double KO of TOX and TOX2 achieved superior tumor control (33). However, a recent study discovered that TOX2 might also regulate central memory differentiation of CAR-T cells (44). TOX2 knockdown in human CAR-T cells decreased the percentage of Tcm cells and their proliferation (44).

### NR4A.

As with TOX, NR4A TFs (NR4A1, NR4A2, NR4A3) act downstream of NFAT to induce exhaustion (33, 45–47). Chen et al. verified elevated NR4A expression in both CD8^+^ CAR-T cells and endogenous tumor-infiltrating lymphocytes with high PD-1 and TIM-3 expression in several solid tumor models (46). Among NR4A members, NR4A3-KO CAR-T cells exhibited the most robust effector functions, with enhanced antitumor cytotoxic activity and augmented cytokine production upon repeated antigen stimulation (48). Compared with single KOs, NR4A–triple KO (NR4A-TKO) CAR-T cells exhibited the greatest antitumor activity in B16-hCD19 and MC38-hCD19 models, characterized by potent effector functions and strong enrichment in accessible chromatin for binding motifs of effector-related TFs (46). Apart from murine CAR-T cells, NR4A-TKO in human CAR-T cells were also resistant to exhaustion, maintaining proliferative capacity and inducing stronger early memory and effector-like effects, which were attributed to enhanced mitochondrial functions and glycolytic systems (17). Of note, positive feedback loops exist among the NFAT, TOX, and NR4A families, where TOX/TOX2 can individually induce NR4A expression, while NR4A can reciprocally induce TOX expression (33).

### BATF and IRF4.

While NFAT and the AP-1 heterodimer Fos-Jun drive T cell activation and differentiation, BATF competes with c-Fos to bind AICEs, suppressing the expression of IL-2 and IFN-γ (11, 49, 50). Single-cell assay for transposase-accessible chromatin with sequencing (ATAC-Seq) demonstrated elevated expression of BATF and IRF4 in terminally exhausted CAR-T cells, consistent with their role in upregulating exhaustion-related genes (35). BATF deletion enhanced CAR-T cell resistance to exhaustion and improved antitumor efficacy (11). BATF and IRF4 can also regulate the persistence and effector functions, whereas BATF knockdown increased the proportion of naive CAR-T cells, and IRF4 knockdown enriched the central memory subsets (11, 35).

**Figure 2:**
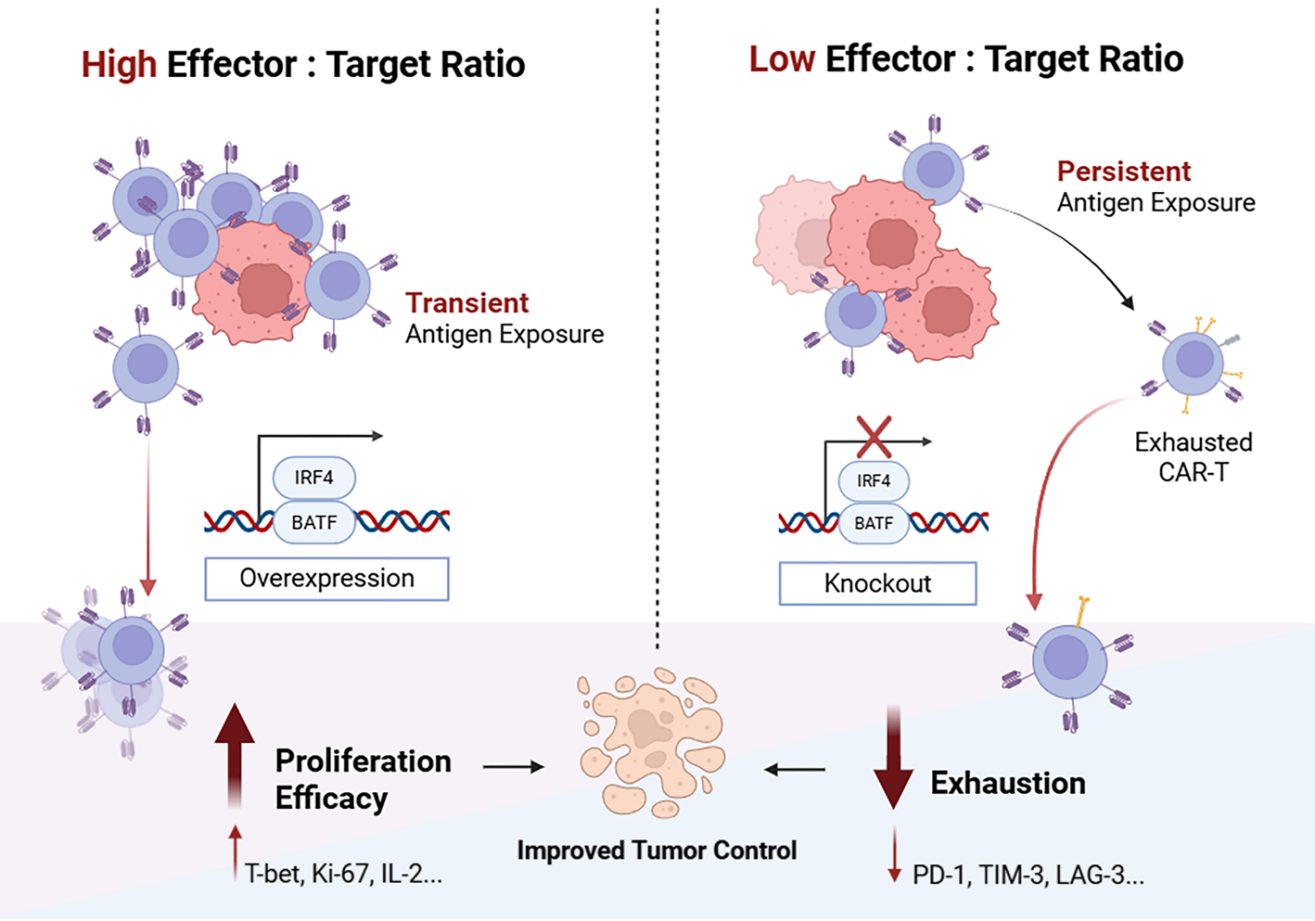
Distinct antitumor effects of BATF and IRF4 manipulation under different effector-to-target ratios. BATF and IRF4 are ambivalent TFs that can contribute to either effector or exhaustion programs in CAR-T cells. Under high effector-to-target (E:T) ratios, overexpression of BATF and IRF4 boosts CAR-T cell proliferation and enhances antitumor efficacy. Conversely, the overexpression of BATF leads to exhaustion, while BATF KO enhances efficacy and alleviates exhaustion under low E:T ratios.

However, several studies yielded contradictory findings. BATF and IRF4 overexpression reduced exhaustion, promoted effector functions, and enhanced tumor-infiltrating CAR-T cells (51). A nonviral pooled knockin (KI) screening platform identified BATF KI as a promoter of T cell fitness (10). The inconsistency can be attributed to the multifaceted roles of BATF and IRF4, which can contribute to either effector or exhaustion programs depending on T cell types, tumor models, and experimental conditions (50, 52, 53). Zhang et al. (11) found that BATF promotes exhaustion under exhaustion-inducing conditions, which are characterized by low effector-to-target (E:T) ratios (ranging from 0.01:1 to 0.4:1) or elevated rounds of tumor stimulation. Conversely, under conditions of high E:T ratio or transient antigen stimulation, BATF promoted T cell proliferation. These controversial results underscore the importance of selecting an appropriate model for investigating TFs with multifaceted functions (Figure 2). Given the immunosuppressive tumor microenvironment, low E:T ratios in vivo are predicted, making exhaustion-inducing in vitro models more reflective of clinical CAR-T cell therapy conditions (11).

### EGR2.

Early growth response 2 (EGR2), a zinc finger TF belonging to the EGR family, acts downstream of TCR-induced NFAT activation and critically regulates T cell development and anergy (54, 55). A recent study showed it also regulates CD8^+^ T cell exhaustion in both chronic lymphocytic choriomeningitis virus and tumor models, maintaining the T cell–exhausted state both transcriptionally and epigenetically (56). Ablation of EGR2 in CAR-T cells not only reduced exhaustion related to the type I IFN pathway but also enhanced proliferation and early memory differentiation during chronic tumor antigen stimulation (36). Clinically, high levels of the EGR2 regulon associated with poor survival, whereas CAR-T cell products enriched with EGR2-KO molecular signatures showed improved clinical outcomes (36).

## TFs related to NK-like exhausted CAR-T cells

Despite the canonical pathway of T cell exhaustion, a recent study demonstrated that CAR-T cells with the 4-1BB costimulatory domain underwent a transition to an NK-like phenotype upon chronic stimulation, with upregulation of NK inhibitory receptors (KLRB1, T cell immunoreceptor with immunoglobulin and ITIM domain [TIGIT] , NKG2A, PD-1) and NK-related proteins (CD56, granulysin) (15). Similar findings were observed in mouse T cells in chronic infection and tumor models (32, 57–59). In addition, clinical data from patients with diffuse large B cell lymphoma receiving CD19-directed CAR-T cells demonstrated a correlation between elevated NK-like CAR-T signatures and therapeutic nonresponse with tumor progression (15, 60). Researchers analyzed their transcriptional programs and discovered a unique profile with elevated expression of several TFs, including inhibitor of DNA binding 3 (ID3) and SRY-box transcription factor 4 (SOX4). KO of these TFs alleviated CAR-T cell exhaustion (Table 2) (15).

### SOX4 and ID3.

An in vitro model of exhausted 4-1BB costimulatory domain (BBz) CAR-T cells identified ID3 and SOX4 as potential regulators of dysfunction via single-cell RNA-Seq (15). SOX4 is an SRY-related box family TF, and ID3 is a helix-loop-helix TF essential for NK cell differentiation from bipotential NK/T progenitors (61–63). Genetic disruption of ID3 and SOX4 can improve CAR-T cell effector functions and reduce NK-like T cells (15). Interestingly, ID3 KO abrogated SOX4 expression, while SOX4 KO only partially reduced ID3 expression, suggesting that ID3 might have additional transcriptional regulators (15).

## TFs associated with stress-induced exhausted CAR-T cells

Solid tumors contain immune cells, stromal cells, blood vessels, extracellular matrix (ECM), and other immune molecules. The ECM in solid tumors exerts a dual role, functioning as both a physical barrier that hinders CAR-T cell infiltration and a catalyst for CAR-T cell exhaustion through biomechanical stress (64, 65). A study using polydimethylsiloxane (PDMS) gels of different stiffnesses revealed that CD8^+^ T cells on high-stiffness PDMS gels are more prone to exhaustion upon repeated antigen stimulation, via the piezo-type mechanosensitive ion channel component 1/Ca^2+^/cAMP-response element binding protein (CREB) axis (66). Of note, mechanical stimulation of CD8^+^ T cells mediates a downstream transcriptional alternation, demonstrating that the modification of mechanical force–related TFs can improve CAR-T cell function (Table 2) (16).

### OSR2.

Odd-skipped related transcription factor 2 (OSR2), a zinc finger TF from the odd-skipped family, is markedly overexpressed in tumor-specific CD8^+^ T cells subjected to biomechanical stress (67). It recruits negative epigenetic regulator histone deacetylase 3 and subsequently downregulates histone H3 lysine 27 acetylation signaling, thereby inhibiting T cell cytotoxicity and exacerbating T cell exhaustion (16, 66). Of note, induction of OSR2 only occurred in exhausted T cells in the tumor microenvironment and not in response to chronic viral infection, which has an absence of substantial mechanical stress (16), suggesting that upregulation of OSR2 may not be a universal mechanism of T cell exhaustion. T cell–specific OSR2-KO mice demonstrated reduced exhaustion of tumor-infiltrating CD8^+^ T cells and CAR-T cells in the MC38 colon tumor mouse model, resulting in enhanced cell proliferation and tumor control. Mechanistically, researchers proposed that OSR2 may synergize with NFAT/TOX signaling to reinforce T cell exhaustion, as elevated levels of TOX and ID3 were observed in OSR2-overexpressing exhausted T cells (16).

## Uncategorized TFs related to CAR-T cell exhaustion

### FOXO3.

A recent study demonstrated that when chronically stimulated, BBz-expressing CAR-T cells present a distinct dysfunctional state compared with CD28-based CAR-T cells (14). BBz CAR-T cells showed low PD-1 and TIGIT expression but high CD62L and CD25 levels. Notably, in dysfunctional BBz CAR-T cells, FOXO3, a Forkhead box O family TF, was overexpressed. FOXO3 plays a crucial role in T cell development and differentiation, and FOXO3 attenuation can promote the formation of memory T cells and prolong their survival (68–70). In comparison with WT BBz CAR-T cells, FOXO3-deficient BBz cells exhibited enhanced antitumor function and prolonged survival in a murine leukemia model. Conversely, FOXO3 overexpression dramatically inhibited BBz cell expansion and led to a loss of tumor control (14).

### ETS1.

Proto-oncogene 1 (ETS1), a member of the E26-transforming specific (ETS) TF family, regulates a range of biological processes in normal cells and tumors (71). Previous research found that mice lacking ETS1 had defects in the development and function of both CD4^+^ and CD8^+^ T cells (72–74). A recent study using single-cell CRISPR screening identified ETS1 as a gatekeeper for precursor exhausted T cells to terminally exhausted T cells (75). Deletion of ETS1 in both OT-1 and CAR-T cells increased effector functions, proliferation, and T cell accumulation in solid tumors (9). Notably, ETS1-deficient T cells manifested increased BATF expression as well as enhanced BATF motif enrichment. Cotargeting both BATF and ETS1 promoted T cell infiltration in the tumor microenvironment and induced superior effector responses (9).

### ETV7.

ETS variant TF 7 (ETV7), an ETS family TF, participates in multiple immune-related pathways and correlates with T cell differentiation, proliferation, and infiltration in various tumors (76–78). A recent study integrated single-cell RNA-Seq and single-cell ATAC-Seq data to identify ETV7 as a key TF of CD8^+^ T cell terminal exhaustion (79). Furthermore, ETV7 expression negatively correlated with patient response and prognosis in anti-CD19 CAR-T cell therapy (79). Knockdown of ETV7 in antimesothelin CAR-T cells enhances antitumor efficacy, with reduced expression of exhaustion markers and increased infiltration in solid tumors (79).

### IKZF.

The IKAROS family zinc finger (IKZF) TFs not only play a pleiotropic role in regulating hematopoiesis but also regulate T cell development and differentiation (80–83). IKZF1 was identified as a major driver of T cell exhaustion in response to repeated antigen stimulation (84). Knockdown of either IKZF1 or IKZF3 in T cells increased expression of several cytokines, including IL-2 (85). Zou et al. demonstrated that IKZF3 KO in anti–human epidermal growth factor receptor 2 (HER2) CAR-T cells increased cytotoxicity both in vitro and in a murine xenograft model of breast cancer (86). AlphaLISA and RNA-Seq analysis revealed upregulated genes involved in cytokine signaling, chemotaxis, and cytotoxicity in IKZF3-KO CAR-T cells. The efficacy of IKZF3 manipulation was also validated on CAR-T cells targeting CD133-positive glioblastoma (86).

However, conflicting results were observed in another study where IKZF1 deletion in OT-1 cells decreased effector functions yet increased the stemness and persistence of cytotoxic lymphocytes in tumors, possibly due to an aberrant quiescent state caused by IKZF1 deficiency (9). These inconsistent outcomes may arise from a distinction between human and murine T cells, and whether the modifications were performed exclusively on CD8^+^ T cells. Further investigation is required to elucidate the roles of IKZF family TFs in T cell development.

### RBPJ.

Recombination signal binding protein for immunoglobulin kappa J region (RBPJ) is a TF known as the downstream effector of Notch signaling that plays an essential role in T cell fate decisions (87, 88). Clinically, high RBPJ expression correlates with CAR-T cell exhaustion and hyporesponsiveness to immunotherapies in patients with cancer (9). Deletion of RBPJ in both OT-1 cells and anti-hCD19 CAR-T cells enhanced proliferation and effector functions in several tumor models (9).

It is noteworthy that RBPJ also interacts with multiple TFs, including IRF1 and BTB domain and CNC homolog 2 (BACH2). KO of RBPJ in T cells increases IRF1 expression, with enhanced effector functions and tumor control. RBPJ expression is regulated by upstream BACH2, and KO of BACH2 increases RBPJ expression (9).

### TFAP4.

Using the modular pooled KI (ModPoKI) approach to screen TF KI combinations at the TRAC locus in T cells, researchers identified transcription factor AP-4 (TFAP4) as a top candidate for enhancing T cell functions (10). TFAP4 is crucial for T cell differentiation, activation, and proliferation upon murine viral infection (89, 90). CD8^+^ T cells from *Tfap4^–/–^* mice failed to maintain their proliferation rate compared with WT cells (89). Moreover, TFAP4 KI of CAR-T cells promoted T cell killing capacity upon repeated antigen stimulation and improved tumor control in a murine leukemia model (10). In particular, CAR-T cells with combined BATF and TFAP4 KI exhibited superior tumor control compared with single KIs, showing a reduction of terminally differentiated Tem cells but increased Tscm and Tcm cells (10).

## TF manipulation to promote CAR-T cell memory differentiation

A significant challenge in CAR-T cell therapy is disease recurrence (4). Consequently, researchers analyzed CAR-T cells from patients who achieved complete disease control and found high expression of memory T cell signatures and enrichment in memory-related genes (26, 27, 29). This discovery led to attempts to select memory T cells for CAR-T cell manufacture (91, 92). However, this approach would substantially increase costs, as memory T cells are only a minor proportion of peripheral blood–derived T cells (93). Therefore, some researchers seek to improve CAR-T cell persistence by modifying the TFs highly expressed in memory T cells (Figure 1 and Table 3) (7, 13, 31).

### FOXO1.

FOXO1, a member of the FOX TF family, switches T cells from an effector profile to a memory phenotype by inhibiting AP-1 and activating TCF1, a TF essential for early memory T cell differentiation (94, 95). Interestingly, 2 papers recently published in *Nature* have both identified FOXO1 as a master regulator of CAR-T cell functions, improving T cell memory and metabolic fitness in various tumor models (12, 13). FOXO1 overexpression promotes a stem-like phenotype in CAR-T cells, with improved mitochondrial fitness, persistence, and antitumor activity in the OVCAR-3 mouse model (13).

Meanwhile, FOXO1 inhibition in CAR-T cells impairs expression of memory-associated genes, leading to an exhaustion-like phenotype (12). In several clinical datasets of anti-CD19 CAR-T cells against leukemia, the activity of FOXO1 in preinfusion CAR-T cells correlates with clinical responses (12).

### BLIMP1.

Encoded by the gene PR/SET domain 1 (*PRDM1*), the TF B-lymphocyte-induced maturation protein 1 (BLIMP1) is essential for T cell exhaustion and terminal differentiation through suppression of TCF1 (96–100). Inspiringly, two recent studies have validated that ablation of *PRDM1* can increase memory CAR-T cell differentiation and enhance their persistence in both hematological and solid tumors (7, 101). Jung et al. knocked out *PRDM1* in prostate-specific membrane antigen (PSMA) CAR-T cells and demonstrated increased TCF1-expressing stem cell–like T cell signatures, suggesting that BLIMP1 inhibits TCF7-dependent stemness (7).

However, PRDM1-KO CAR-T cells failed to eradicate tumors due to attenuated effector functions, marked by reduced cytolytic molecules and increased expression of exhaustion-related markers (7, 101). After chronic antigen stimulation, PRDM1-KO CAR-T cells upregulated classical exhaustion-related TFs (NR4A, TOX, IRF4), showing a negative epigenetic feedback program of NFAT-driven T cell dysfunction. Notably, dual ablation of *PRDM1* and *NR4A3* successfully counteracted T cell exhaustion and exhibited superior antitumor capacity, with increased frequency of stem cell–like TIM-3^–^TCF1^+^CD8^+^ T cells in both tumor and peripheral blood (7). These results highlight the importance of understanding the complex regulatory network of TFs, which enables more effective combinatorial manipulation.

### RUNX3.

Runt related transcription factor 3 (RUNX3) is a key regulator of CD8^+^ T cell development and tissue-resident memory T cell (Trm) generation (102). Several studies have shown that CAR-T cells with RUNX3 overexpression can overcome the limited efficacy and exhibit enhanced antitumor effects and improved persistence in vivo (18, 103). In a hepatocellular carcinoma model, GPC3-BBz CAR-T cells with RUNX3 overexpression exhibited better tumor control, with increased CAR-T cell numbers in blood and tumor tissue, along with a higher proportion of Tem cells (18). Mechanistically, these CAR-T cells resisted activation-induced cell death in solid tumors, leading to longer persistence and durable antitumor responses (18). In phase I clinical trials (NCT03980288), GPC3 CAR-T cells with RUNX3 overexpression showed an objective response rate of 16.7% and a disease control rate of 50% (104). While CRS developed in all patients (grade 3 CRS incidence: 50%, *n* = 3/6), these patients recovered following tocilizumab treatment, with or without glucocorticoids. These preliminary findings highlight the need for further clinical investigation (104).

Consistently, another study validated the efficacy of RUNX3-overexpressing CAR-T cells in urinary bladder tumors with more naive cells. These cells showed reduced cytokine release, lowering GVHD risk and enhancing systemic safety (103).

As RUNX3 is involved in generating Trm cells, Tang et al. combined RUNX3 overexpression with ex vivo AKT inhibition to generate CAR-T cells with both Trm and Tcm characteristics (105). While AKT inhibition alone induced a CD62L^+^ central memory–like phenotype with impaired tumor-resident and effector functions in CAR-T cells, AKT inhibition in combination with RUNX3 overexpression improved cytotoxic potential and tumor infiltration in a pancreatic ductal adenocarcinoma model (105).

### FOXP1.

FOXP1 is essential for naive T cell quiescence and acts as a hub TF in the stem-like CAR-T cell network (31, 106). It promotes T cell stemness and limits T cell differentiation from the stem-like subset to the effector-like subset. Deletion of FOXP1 after T cell activation significantly reduced the stem-like subset with increased frequencies of effector-like subset and NK-like subset, showing impaired antitumor activity in B16-CD19 mice (31). In another study, FOXP1 deletion in *Cd4-Cre* mice enhanced T cell effector functions and proliferation, prolonging survival in an ovarian cancer model (107). The controversial results above showed different effects of FOXP1 on the antitumor activity of T cells before and after T cell activation, which will be discussed later.

### KLF2 and KLF7.

Kruppel-like factor 2 (KLF2) is highly expressed in naive T and memory T cells and is crucial for T cell quiescence and survival (108). Single-cell transcriptomic and epigenetic analysis have identified KLF2 as a hub TF in CAR-T cell differentiation. KLF2 expression increased the proportion of effector CD8^+^ T cell and prevented terminal exhaustion by negatively regulating TOX expression (31). Loss of KLF2 led to increased TOX expression, impaired effector differentiation, and increased exhaustion program, with reduced antitumor capacity against B16-CD19 tumor (31).

KLF7, another KLF family member, is essential for neuronal morphology and carcinogenesis (108, 109). Histone mark analysis indicates higher KLF7 expression correlated with superior CAR-T therapy outcomes in patients (110). Overexpressing KLF7 in anti-CD19 CAR-T cells led to a less differentiated CCR7^+^CD45RA^–^ phenotype and increased T cell proliferation (110).

## Factors influencing TF manipulation outcomes

Interestingly, studies have reported conflicting results when modifying the same TF in CAR-T cells because of different experimental conditions. For example, both overexpression and knockdown of BATF and IRF4 have been shown to improve CAR-T efficacy. Indeed, the regulation of TFs in CAR-T cells is influenced by several factors, including the T cell activation status, CAR structure, TF expression level, and in vitro experimental conditions. Here we summarize some critical aspects influencing TF manipulation outcomes.

## T cell activation state

The TF modulation results are associated with T cell activation state. Deletion of FOXP1 in activated murine T cells impairs antitumor activity in B16-CD19 mice (31), while *Foxp1^–/–^* T cells have better effects in an ovarian tumor model (107). Similarly, while FOXO1 overexpression promoted CAR-T memory phenotypes, FOXO1 inhibition in T cells without conventional preactivation generated more Tscm cells (111). These studies collectively demonstrate that the effect of TFs in CAR-T cells is associated with the activation status of T cells, and further understanding of TF regulation mechanisms is needed.

## CAR structure

The costimulatory domain of the CAR structure influences TF effects. For example, FOXO3 KO improves antitumor activity in CAR-T cells with a 4-1BB costimulatory domain but not in T cells with CD28 domain. This result was aligned with the transcriptional and epigenetic divergences between costimulatory subtypes (14).

In addition, while c-Jun overexpression resisted T cell exhaustion in several studies, it failed to enhance DNAX-activating protein (DAP) CAR-T cell efficacy (112). This discrepancy may result from the reliance of c-Jun effects on downstream CD3ζ signaling in conventional CAR structure, as opposed to the distinct inner signaling components of DAP CAR.

## TF expression level

Currently, TF overexpression mainly involves conventional viral and nonviral KI approaches. While viral vectors can deliver CAR and TF constructs with higher efficiency, their random integration can lead to variable TF expression levels depending on the integration site. In addition, viral vector copy numbers can affect transgene expression (113, 114). Conversely, nonviral ModPoKI screening enables precise single-copy integration at a specific genomic site, ensuring consistent transgene expression (10).

Blaeschke et al. revealed distinct effects between nonviral KI and retroviral transduction (10). CAR-T cells overexpressing TFAP4 via viral transduction failed to enhance fitness and cytokine secretion compared with TRAC-targeted KI cells (10). These findings underscore the importance of TF expression levels for CAR-T cell functions. Future investigations should focus on maintaining genetic modification in the same genomic context.

## In vitro experimental conditions

In addition to the CAR structure, an appropriate E:T ratio is also crucial for studying antitumor efficacy in vitro. At high E:T ratios (≥2:1), BATF overexpression enhanced tumor lysis, while under low E:T ratios (≤0.4:1) or repetitive tumor stimulation, BATF depletion reversed CAR-T exhaustion and enhanced cytotoxicity (11). Similarly, TFAP4 KI exhibited only a mild effect in a single-stimulation screening but a more pronounced function when exposed to repetitive antigen stimulation (10). These controversial results highlight the importance of choosing appropriate models to investigate TFs with multiple functions. Research suggests that in the tumor microenvironment, the E:T ratio is likely to be low, and CAR-T cells tend to be in an exhausted state (11). Therefore, choosing experimental conditions with low E:T ratios or repeated tumor stimulation in vitro may better mimic the in vivo tumor microenvironment (11).

## Perspectives

### Multiple-TF manipulation for better CAR-T cells.

**Figure 3:**
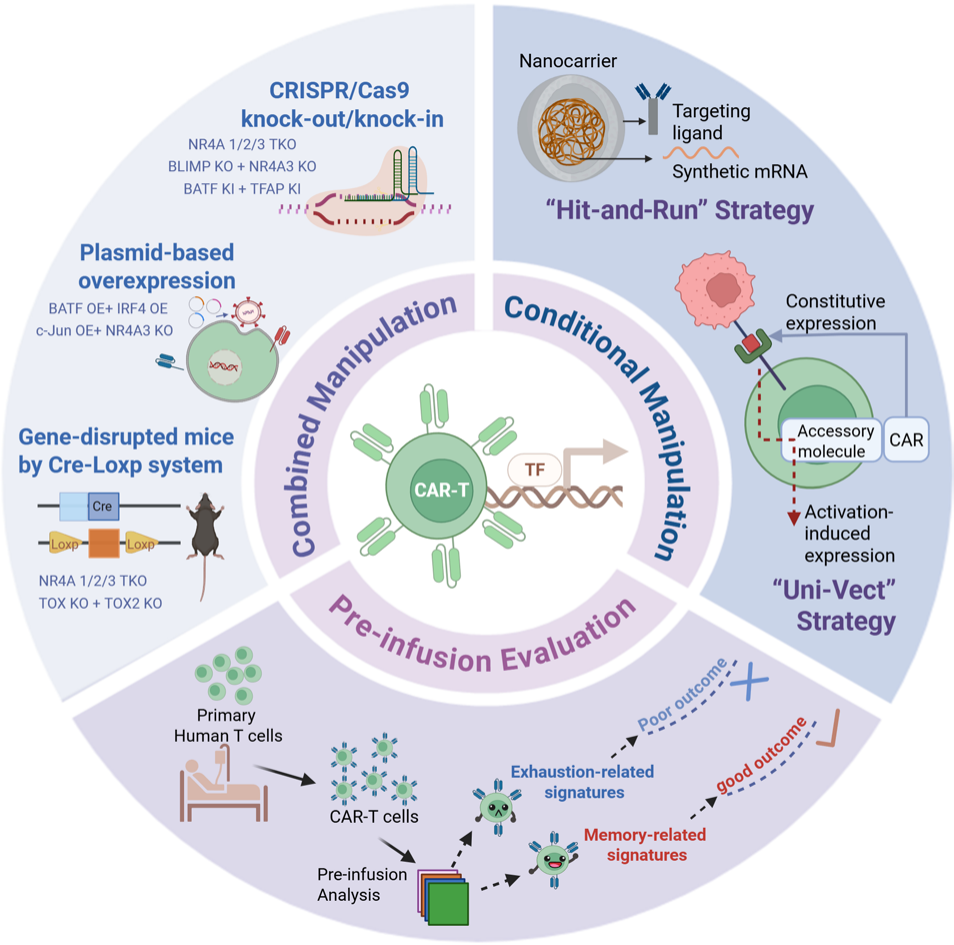
Strategies for TF manipulation to augment CAR-T cell antitumor activity. This figure provides a perspective on the optimization of TF manipulation. Current combined manipulation includes plasmid-based overexpression (OE), CRISPR/Cas9 knockout (KO)/knockin (KI), and Cre-*loxp* gene-disrupted mice. Conditional manipulation is another strategy, including (a) “Hit-and-Run” strategy, which utilizes T cell–targeted nanocarriers to transiently express TFs, and (b) “Uni-Vect” system, which induces TF expression only when NFAT-driven signaling is activated. Analyzing the transcriptomic profile of preinfusion CAR-T cells may predict clinical outcomes and patient survival in CAR-T cell therapy. TKO, triple knockout.

Although most current studies focus on single-TF modification in CAR-T cells, there are also studies on manipulating multiple TFs simultaneously to achieve better function (Figure 3). For instance, double KO of TOX1 and TOX2 exhibited superior tumor control in solid tumors (33). Similarly, the TKO of the NR4A family (NR4A1, NR4A2, and NR4A3) exhibited the most effective antitumor activity compared with single KOs (17, 46). Researchers have also attempted to manipulate several TFs from different families on the same regulatory pathway, such as the NFAT-TOX/NR4A axis. The combination modification of NR4A3 KO and c-JUN overexpression produced better CAR-T cell expansion and superior antitumor efficacy in vivo compared with c-JUN overexpression alone (48). Another example is the success of combined BLIMP1 and NR4A3. Single BLIMP KO led to enhanced expansion but impaired effector functions resulting from the upregulation of exhaustion-related TFs (NR4A, TOX, IRF4); however, simultaneous KO of both BLIMP and NR4A3 enhanced proliferation and reduced exhaustion during chronic antigen exposure (7).

In addition to KO of multiple TFs, researchers have explored overexpression of multiple TFs in CAR-T cells (Figure 3). Overexpression of both BATF and IRF4 via retroviral transduction has achieved reduced exhaustion and enhanced effector functions in CAR-T cells (51). Another study employed nonviral ModPoKI screening, which is combinatorial KI of BATF and TFAP4, and demonstrated effective tumor control against Nalm-6 leukemia with GD2 expression (10).

Although several studies have achieved multigene editing via the CRISPR/Cas9 system or other methods (115–117), there are still many challenges, including unpredictable editing efficiency and genotoxicity risks (118–120). Manipulation of multiple TFs necessitates a comprehensive understanding of TF regulatory networks, along with development of technologies to enhance efficiency and safety.

### Conditional TF manipulation for safety.

Engineering TFs in CAR-T cells carries several risks. For instance, c-Jun is a homolog of the viral oncogene vJun, and its overexpression has been reported in several types of tumors (8). BATF and BATF3 have also been reported to potentially drive unrestrained proliferation (121–124). To mitigate these risks, partial or transient TF manipulation may be a safer alternative to constant expression (Figure 3) (125–127). For instance, mRNA nanocarriers were used to transiently express FOXO1. After coculture with FOXO1 mRNA-loaded nanoparticles, CAR-T cells exhibited and increased proportion of Tcms and enhanced antitumor efficacy against B cell lymphoma (126). Another group established an inducible platform called Uni-Vect, which requires enough NFAT-driven signaling for TF expression. Developing 2 Uni-Vect systems to combine a constitutive HER2-targeting CAR with NFAT-inducible FOXO1 or TCF7, researchers validated improved proliferation and persistence of CAR-T cells in ovarian tumors (127).

Of note, some TFs are vital for T cell development and functions, and their KO may result in deleterious side effects. For instance, Egr2 deficiency in T cells causes lupus-like autoimmune disease (55). Additionally, TOX is crucial for the long-term maintenance of T cell function, and its deletion impaired antitumor efficacy under chronic stimulation (128, 129). Consequently, there is an urgent need to develop strategies to achieve transient TF effects in CAR-T cells.

### Evaluating TF-related signatures to predict CAR-T clinical efficacy.

Currently, there are seven CAR-T cell products approved by the FDA and hundreds of ongoing clinical trials. Before administration, CAR-T cell products undergo a series of quality control tests to confirm identity, purity, safety, and potency (130, 131). Based on clinical results, additional parameters could be considered for more concise preinfusion evaluation. For instance, preinfusion of CAR-T cells with higher expression of memory-like signatures exhibits greater potential for complete tumor control (26, 27, 29). In addition, the transcriptomic profile of preinfusion CAR-T cells also correlates with clinical prognosis (13, 36). A recent study found that FOXO1 activity in CAR-T cells was associated with clinical outcomes and survival (12). Another study determined that a higher EGR2-related signature correlates with poor survival in patients (36). These results raise the question: Can we identify transcriptional programs of CAR-T cells modulated by specific TFs and use them to evaluate preinfusion CAR-T cell products, thereby predicting CAR-T therapy efficacy in patients (Figure 3)? Furthermore, current analysis and screening approaches for candidate TFs are based on simple functional metrics, such as a memory-like signature. More comprehensive assessments, involving both functional and phenotypic effects, could be incorporated.

## Conclusions

Although CAR-T cell therapy has shown remarkable success for treating hematological tumors, its application in solid tumors remains challenging. As TFs are critical for CAR-T cell differentiation and therapeutic efficacy, this Review summarizes current studies on TF manipulation in CAR-T cells, highlighting the improved antitumor capacity of knocking out exhaustion-associated TFs or overexpression of memory-associated TFs. TFs are part of a complex regulatory network, and their effects on CAR-T cells are influenced by several factors, such as T cell activation state, CAR design, TF expression level, and in vitro culture conditions. With deeper insight into TF regulatory networks, we can optimize CAR-T cell performance through manipulation of multiple TFs and conditional TF expression to optimize performance. Moreover, analyzing TF-related signatures to evaluate the clinical efficacy of CAR-T therapy holds great promise for clinical translation.

## Author contributions

JS and RC contributed to conception and manuscript design. RC and LC wrote the manuscript. RC and LC drew the figures and tables. YT, X Shen, YW, PT, X Shentu, and JS participated in the revision of the manuscript. TS and JS were involved in funding acquisition. All the authors discussed and approved the final manuscript.

## Figures and Tables

**Table 1:**
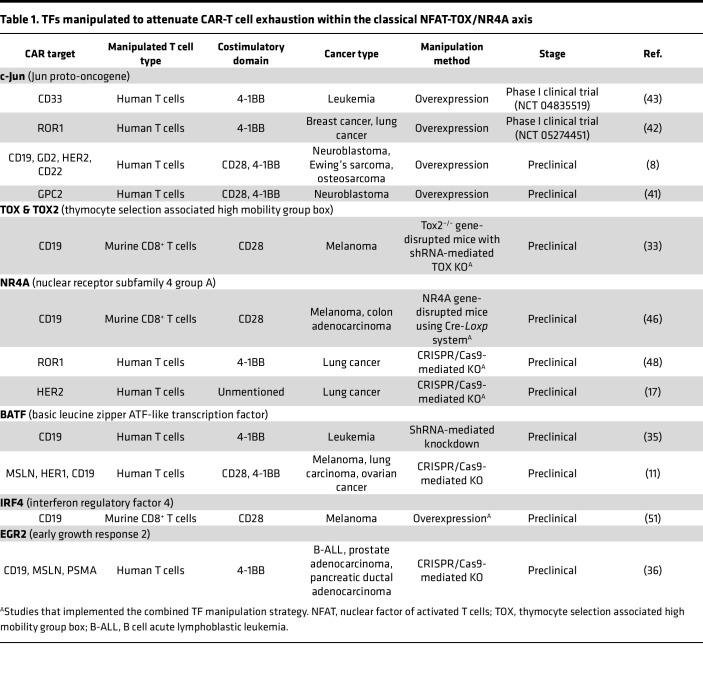
TFs manipulated to attenuate CAR-T cell exhaustion within the classical NFAT-TOX/NR4A axis

**Table 2:**
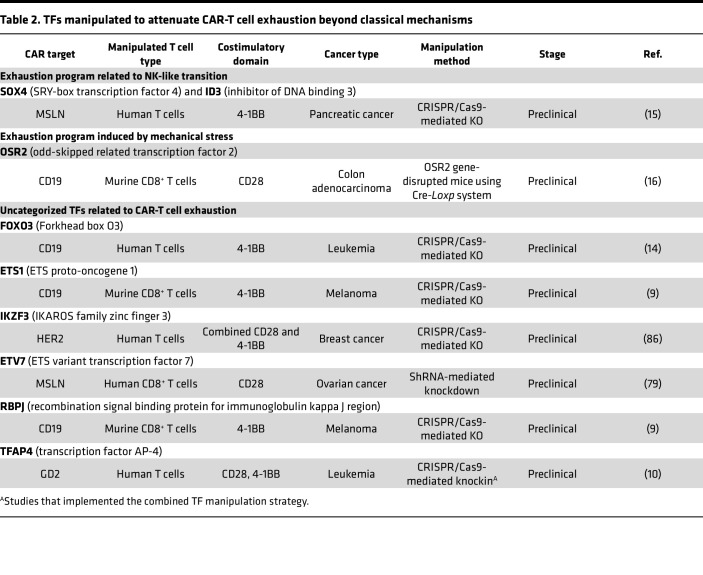
TFs manipulated to attenuate CAR-T cell exhaustion beyond classical mechanisms

**Table 3:**
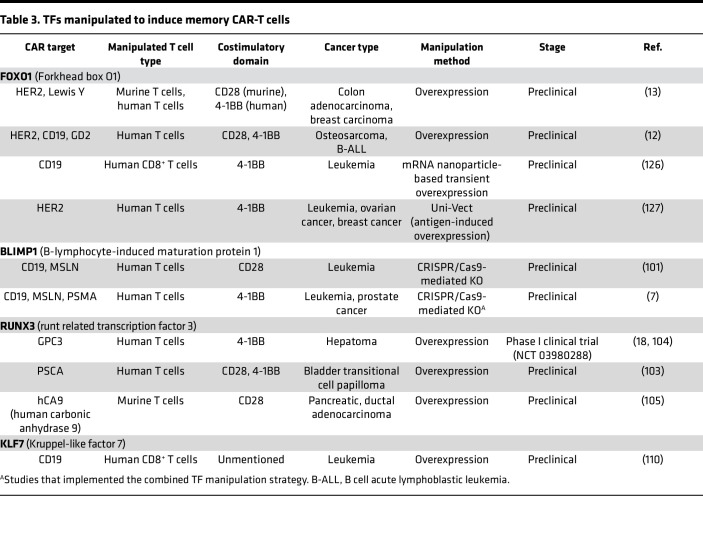
TFs manipulated to induce memory CAR-T cells

## Version 1 07/22/2025

Electronic publication
